# Complete chloroplast genome of *Lilium ledebourii* (Baker) Boiss and its comparative analysis: lights into selective pressure and adaptive evolution

**DOI:** 10.1038/s41598-022-13449-x

**Published:** 2022-06-07

**Authors:** Morteza Sheikh-Assadi, Roohangiz Naderi, Mohsen Kafi, Reza Fatahi, Seyed Alireza Salami, Vahid Shariati

**Affiliations:** 1 Department of Horticultural Science, Faculty of Agricultural Science and Engineering, University of Tehran, Karaj, Iran; 2 NIGEB Genome Center, National Institute of Genetic Engineering and Biotechnology, Tehran, Iran

**Keywords:** Classification and taxonomy, Data processing, High-throughput screening, Phylogeny, Sequence annotation, Plant sciences, Plant evolution, Plant genetics, Molecular evolution, Phylogenetics

## Abstract

*Lilium ledebourii* (Baker) Boiss is a rare species, which exhibits valuable traits. However, before its genetic diversity and evolutionary were uncovered, its wild resources were jeopardized. Moreover, some ambiguities in phylogenetic relationships of this genus remain unresolved. Therefore, obtaining the whole chloroplast sequences of *L. ledebourii* and its comparative analysis along with other *Lilium* species is crucial and pivotal to understanding the evolution of this genus as well as the genetic populations. A multi-scale genome-level analysis, especially selection pressure, was conducted. Detailed third‑generation sequencing and analysis revealed a whole chloroplast genome of 151,884 bp, with an ordinary quadripartite and protected structure comprising 37.0% GC. Overall, 113 different genes were recognized in the chloroplast genome, consisting of 30 distinct tRNA genes, four distinct ribosomal RNAs genes, and 79 unique protein-encoding genes. Here, 3234 SSRs and 2053 complex repeats were identified, and a comprehensive analysis was performed for IR expansion and contraction, and codon usage bias. Moreover, genome-wide sliding window analysis revealed the variability of *rpl*32-*trn*L-*ccs*A, *pet*D-*rpo*A, *ycf*1, *psb*I-*trn*S-*trn*G, *rps*15-*ycf*1, *trn*R, *trn*T-*trn*L, and *trn*P-*psa*J-*rpl*33 were higher among the 48 *Lilium* cp genomes, displaying higher variability of nucleotide in SC regions. Following 1128 pairwise comparisons, *ndh*B, *psb*J, *psb*Z, and *ycf*2 exhibit zero synonymous substitution, revealing divergence or genetic restriction. Furthermore, out of 78 protein-coding genes, we found that *acc*D and *rp*l36 under positive selection: however, at the entire-chloroplast protein scale, the *Lilium* species have gone through a purifying selection. Also, a new phylogenetic tree for *Lilium* was rebuilt, and we believe that the *Lilium* classification is clearer than before. The genetic resources provided here will aid future studies in species identification, population genetics, and *Lilium* conservation.

## Introduction

In keeping with APG IV, Liliales are made up of about 1500 species divided into 10 families that have been categorized by various authors^1^. The Liliales order is constantly being studied botanically, as variously amended family circumscriptions^2–4^. Some of the changes in Liliales, for example, include^4–6^: (1) Petermanniaceae has been recognized as a family, while it was within Colchicaceae; (2) Luzuriagaceae has been placed in Alstroemeriaceae; and (3) Corsiaceae has recently been placed in Liliales. Liliaceae contains 15 genera and around 900 species^7^. The family’s classification has shifted significantly as a result of modern molecular phylogenetic analysis^1,8,9^. *Lilium* is a genus in Liliaceae, which contains circa 100 species^10^. This genus classification has historically been obscurant. Based on morphological characteristics, several classifications of *Lilium* have been suggested. This genus was classified into seven sections relying on 13 morphological characteristics^11^, which have primarily been applied to distinguish *Lilium* species and *Lilium* phylogeny exploration^12^. Nevertheless, this type of species classification based on morphological traits is ofttimes dynamic and untrustworthy and is frequently influenced by environmental circumstances; that is why there is some disagreement about Comber’s classification. Therefore, the Comber’s classification verification has been checked by molecular phylogenetic investigations^13–16^. Additionally, Kim et al.^17^, Du et al.^18^, and Kim et al.^12^ evaluated *Lilium* phylogeny using 9, 16, and 28 species, respectively, which somewhat resolved the phylogenetic relationships, but some ambiguities still remain. For example, due to sampling restrictions for Lophophorum and Nomocharis, the position of *L. distichum* has not been clarified enough. Kim et al.^12^, according to their results, pointed out that the position of *L. candidum* is uncertain, and more sampling is required to resolve it.

Furthermore, due to the sampling and hybridization of these species to produce today’s lilies, understanding wild lilies is essential for achieving breeding program goals^19^. *L. ledebourii*, called Susan-e Chelcheragh (SCh) in Persian, is a rare species in the genus *Lilium*. It has only been seen in Iran and Azerbaijan. Due to uncontrolled grazing and poaching, it is extremely endangered, now being protected only in a small area of Damash village^20,21^. *L. ledebourii* exhibits valuable traits, including attractive white flower^20^, a high number of flowers, usually 2–15^22^ and even up to 24 (personal observations of the author, 2016), the sweet fragrance^23^ ^quoted by^^24^, an excellent vase-life, a vigorous growth, a good tolerance to low light density and low temperatures^25^. These make it no less beautiful than the commercial species. However, despite being highly valuable and, more importantly, endangered, has not only little effort has been made to use the species in population genetic studies, but also its rightful position among lilies is unclear.

DNA barcoding is one of the most efficient methods for characterizing and classifying various organisms at the species and genus levels^26^. One of the research hotspots for DNA barcode screening is chloroplast (cp), which can be employed as a super-barcode to solve the classification problem in phylogenetic studies and species identification^27,28^. Chloroplasts, the energy generators of plant cells, ensure life on the earth^29,30^. This vital organ serves as a signaling hub in the cell, releasing a diverse range of signals that adjust a well-regulated and proper reaction to any condition^31^. The contents, structure, and gene organization of a chloroplast genome are more strictly conserved than a nucleic genome^32^. It has a much lower substitution rate than a nucleic genome, and the substitution rate is even lower in two inverted repeat regions^33^. Noteworthy, information included in chloroplast genomes, as well as their almost nonrecombinant traits^34^, maternal transmission^35^, have made the chloroplast genome a good source for searching for clues about the origins of populations as well as for phylogenetic reconstructions, thereby clearing the ambiguities present in the evolutionary relationships^36^. Hence, today's strategies for discovering plant molecular phylogeny rely profoundly on cp genome sequences. Additionally, because of advances in next-generation sequencing technology, chiefly third-generation sequencing such as PacBio, leaning on single-molecule real-time (SMRT), which produces reads > 10 kb, the decoding of chloroplast genomes has been accelerated^37,38^.

Other advantages of chloroplasts include using mutation hotspot sites, and the single sequence repeats to aid population genetics and species identification^39^. The selection pressure that a species face during evolution is another fascinating aspect of chloroplast genome analysis, which divulged the impact of various environmental pressures on cp genomes when it comes to long-term evolution^40^. Recent studies have discovered a slew of positive selection genes e.g., the *acc*D in Ipomoea^41^, and *pet*G, *rpl*36, and *atp*B in Aquilegia^42^, as well as purifying selection at cp genome-scale in *Stauntonia*^43^, but we know very little about it in *Lilium* at the gene-level and nothing at the cp genome-level.

This study, employing PacBio platform, reports the whole chloroplast genome of *L. ledebourii*, a precious endangered species. In addition, using the genome data, we conducted a multi-scale genome-level analysis among this species, newly unemployed (at the genome-scale) species, and other *Lilium* species. In particular, for the first time, we presented a comprehensive analysis of the selection pressure between *Lilium* species at both the gene-level and genome-level. This study covered overlooked topics in previous studies as much as possible. Lastly, employing the richer taxon sampling, we rebuilt a new phylogenetic tree for *Lilium* based on the whole cp genome, and we believe that we have given more resolution to the *Lilium* classification than in earlier studies. This article may be the primary cornerstone for future molecular studies and genetic improvement of *L. ledebourii*.

## Results

### The chloroplast genome features of *L. ledebourii*

**Figure 1:**
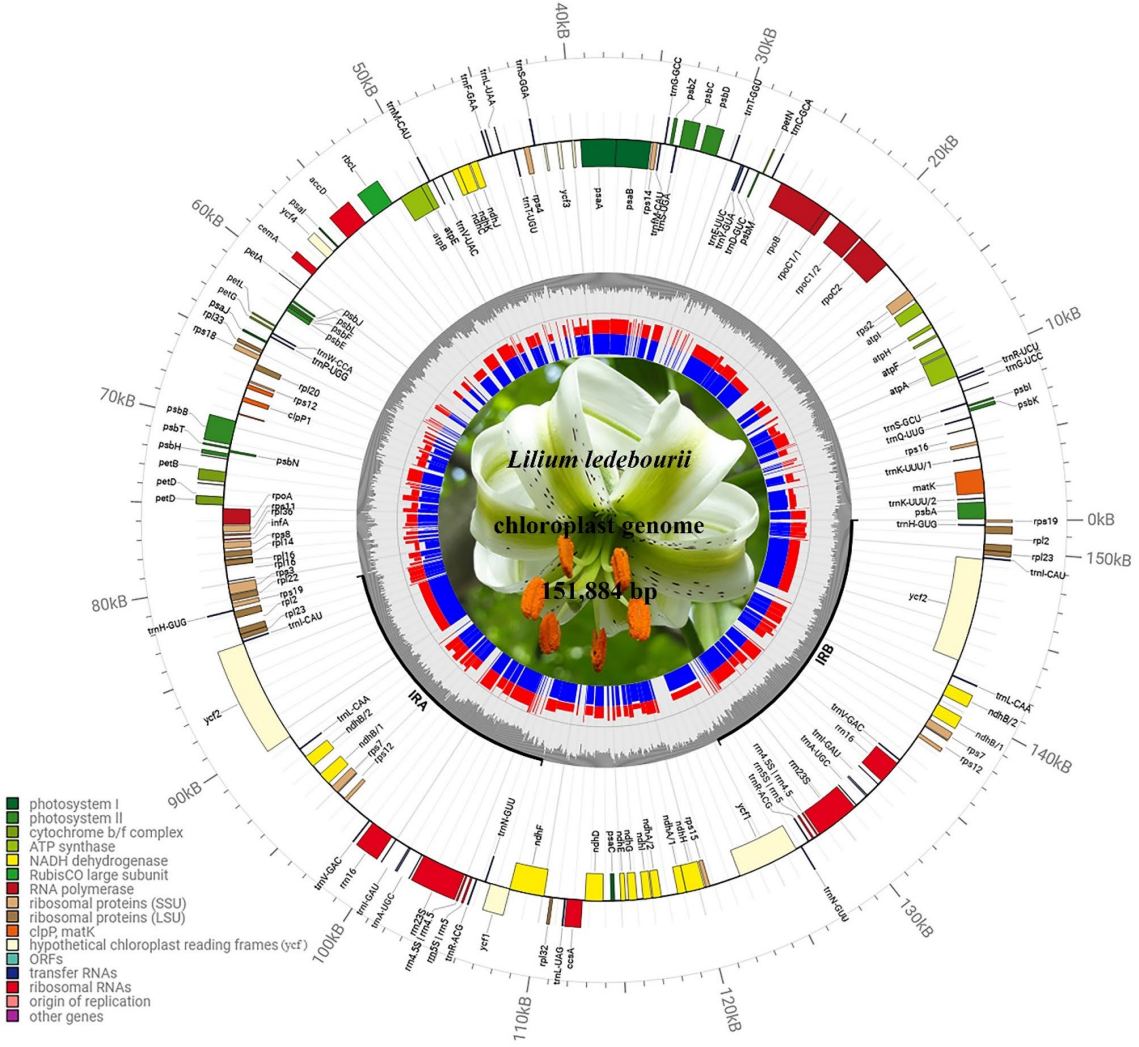
The chloroplast genome map of *L. ledebourii*. Transcriptional directions are represented on the circle's inside (clockwise) and outside (counterclockwise). Genes are color-coded according to their functional groups.

The *L. ledebourii* cp genome, as typical, showed the ordinary quadripartite and protected structure with 151,884 bp in length. The two regions of LSC (81,412 bp) and SSC (17,620 bp) were present in the *L. ledebourii* genome, separated by two inverted repeats (26426 bp), IRa and IRb (Fig. 1). *L. ledebourii* cp chloroplast has 37.0% GC content, with IRs having the highest (42.5%) and SSC having the lowest (37.5%). Overall, 113 different genes were recognized in the SCh chloroplast genome, consisting of 30 distinct tRNA genes, four distinct ribosomal RNAs (4.5S, 5S, 16S, and 23S) genes, and 79 unique protein-encoding genes (Fig. 1, Table 1). Primarily based on their features, all genes are classified into five main categories (Table 1). Among 113 genes, most of the genes happen without another copy in the LSC or SSC regions, whereas 20 are copied within the IR regions. In addition, 18 genes in the *L. ledebourii* chloroplast genome contained introns, including 12 proteins encoding genes (*rps*12, *rps*16, *rpl*2, *rpl*16, *pet*B, *pet*D, *ndh*A, *ndh*B, *clp*P, *ycf*3, *rpo*C1, and *atp*F) and 6 tRNAs (*trn*G-UCC, *trn*L-UAA, *trn*K-UUU, *trn*V-UAC, *trn*A-UGC, and *trnI*-GAU), of which 18 genes with a single intron and two genes (*clp*P and *ycf*3) with two introns. *inf*A was interpreted as a pseudogene. Trans-splicing was observed in the *rps*12 gene, with the 5′ exon positioned in the LSC region and the intron and 3′ exon positioned in the IR regions (Fig. 1, Table 1).

**Table 1:** Gene content and functional classification of *L. ledebourii* chloroplast genome.

Category	Gene group	Gene name
Photosynthesis pathways	ATP synthase	*atp*A, *atp*B, *atp*E, *atp*F*, *atp*H, *atp*I
	NADH-dehydrogenase	*ndh*A*, *ndh*B*, *ndh*C, *ndh*D, *ndh*E, *ndh*F, *ndh*G, *ndh*H, *ndh*I, *ndh*J, *ndh*K,
	Cytochrome b/f complex	*pet*A, *pet*B*, *pet*D*, *pet*G, *pet*L, *pet*N
	Photosystem I	*psa*A, *psa*B, *psa*C, *psa*I, *psa*J
	Photosystem II	*psb*A, *psb*B, *psb*C, *psb*D, *psb*E, *psb*F, *psb*H, *psb*I, *psb*J, *psb*K, *psb*L, *psb*M, *psb*N, *psb*T, *psb*Z
	Rubisco	*rbc*L
Transcription and translation related genes	DNA-dependent RNA polymerase	*rpo*A, *rpo*B, *rpo*C1*, *rpo*C2
Ribosomal proteins	Large subunit of ribosomal proteins	*rpl*2*, *rpl*14, *rpl*16*, *rpl*20, *rpl*22, *rpl*23, *rpl*32, *rpl*33, *rpl*36
	Small subunit of ribosomal proteins	*rps*2, *rps*3, *rps*4, *rps*7, *rps*8, *rps*11, *rps*12*^a^, *rps*14, *rps*15, *rps*16*, *rps*18, *rps*19
RNA genes	Ribosomal RNA	*rrn*4.5 (× 2), *rrn*5 (× 2), *rrn*16 (× 2), *rrn*23 (× 2)
	Transfer RNA	*trn*A-UGC* (× 2), *trn*C-GCA, *trn*D-GUC, *trn*E-UUC, *trn*F-GAA, *trn*fM-CAU, *trn*G-GCC, *trn*G-UCC*, *trn*H-GUG (× 2), *trn*I-CAU (× 2), *trn*I-GAU* (× 2), *trn*K-UUU*, *trn*L-CAA (× 2), *trn*L-UAA*, *trn*L-UAG, *trn*M-CAU, *trn*N-GUU (× 2), *trn*P-UGG, *trn*Q-UUG, *trn*R-ACG (× 2), *trn*R-UCU, *trn*S-GCU, *trn*S-GGA, *trn*S-UGA, *trn*T-GGU, *trn*T-UGU, *trn*V-GAC (× 2), *trn*V-UAC*, *trn*W-CCA, *trn*Y-GUA
Other genes	Maturase K	*mat*K
	Subunit of acetyl-CoAcarboxylase	*acc*D
	C-type cytochrome synthesis gene	*ccs*A
	Envelope membrane protein	*cem*A
	ATP-dependent protease subunit P	*clp*P**
	Translational initiation factor	*inf*A^ψ^
	Conserved hypothetical open reading frames	*ycf*1, *ycf*2, *ycf*3**, *ycf*4

*Gene with one intron; **gene with two introns; (×2) duplicated gene; ^a^trans-spliced gene; ψ: pseudogene.

### Genome comparison: boundaries regions and divergence hotspot

**Figure 2:**
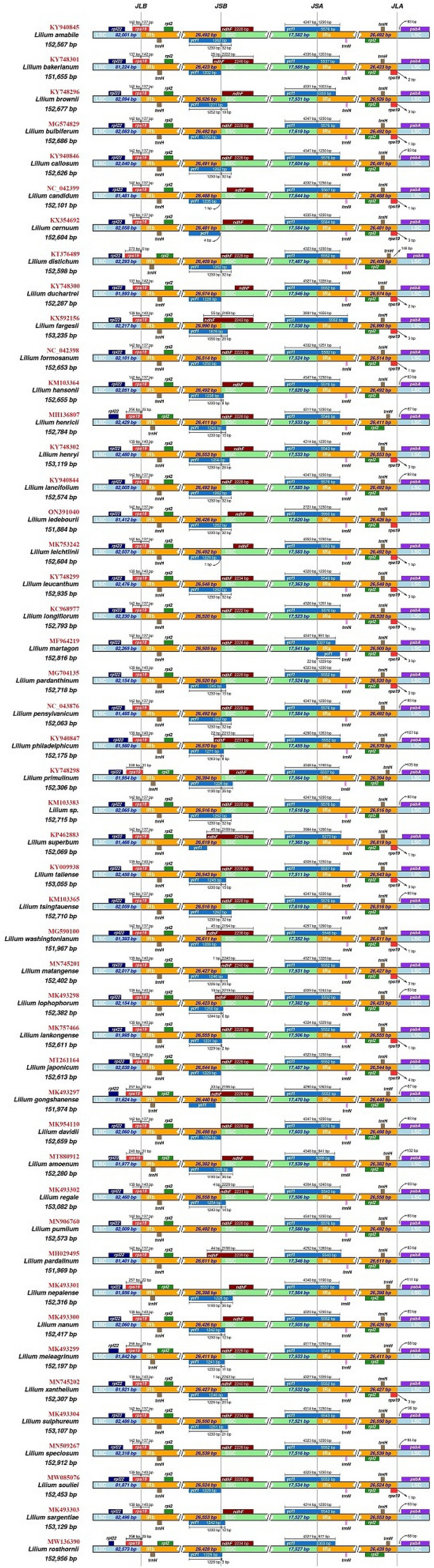
Comparison of the junction positions of LSC, SSC, and IR regions among the among 48 Lilium cp genomes. The red identifiers represent the GenBank accession number of each species.

The *Lilium* cp genomes size differed between 151,655 bp in *L. bakerianum* and 153,235 bp in *L. fargesii*. The expansion and contraction variability in IR/SC junction regions, which were typical phenomena in the plant species evolutionary scrutiny, were evaluated by comparing the border regions and adjacent genes of *Lilium* chloroplast genomes. In this study, the chloroplast genomes of *Lilium* species demonstrated slightly visible junction variation in the IRa/LSC and IRb/SSC boundaries, despite the gene number and gene content being conserved (Fig. 2).

In all *Lilium* cp genomes, IR regions were found to have nearly the same size (26,382 bp to 26,990 bp). *L. fargesii* had the largest IR region expansion, which ended at the *rps*19 gene. In all *Lilium* cp genomes, the *ycf*1 gene was placed in the IR/SSC boundary regions, leading to an incomplete gene duplication within IRs, and the IR/LSC junction site was placed in the *rps*19 gene. In most *Lilium* cp genomes, the rps19 gene was observed in the IRb region around 137 bp to 142 bp far from the JLB boundary, except for *L. distichum* (6 bp); *L. gongshanense* and *L. nepalense* (22); *L. henricii*, *L. meleagrinum*, and *L. rosthornii* (23 bp); and *L. amoenum* and *L. primulinum* (31 bp): it was found significantly lower than the other species. The *ndh*F gene in most cp genomes (39/48) was positioned inside the SSC location. However, in nine species, including *L. bakerianum* 25 bp, *L. fargesii* 55 bp, *L. gongshanense* 33 bp, *L. lophophorum* 19 bp, *L. pardalinum* 44 bp, *L. philadelphicum* 22 bp, *L. regale* 4 bp, *L. superbum* 45 bp, and *L. washingtonianum* 45 bp, the *ndh*F gene was discovered somewhat extended in the IRb (Fig. 2).

**Figure 3:**
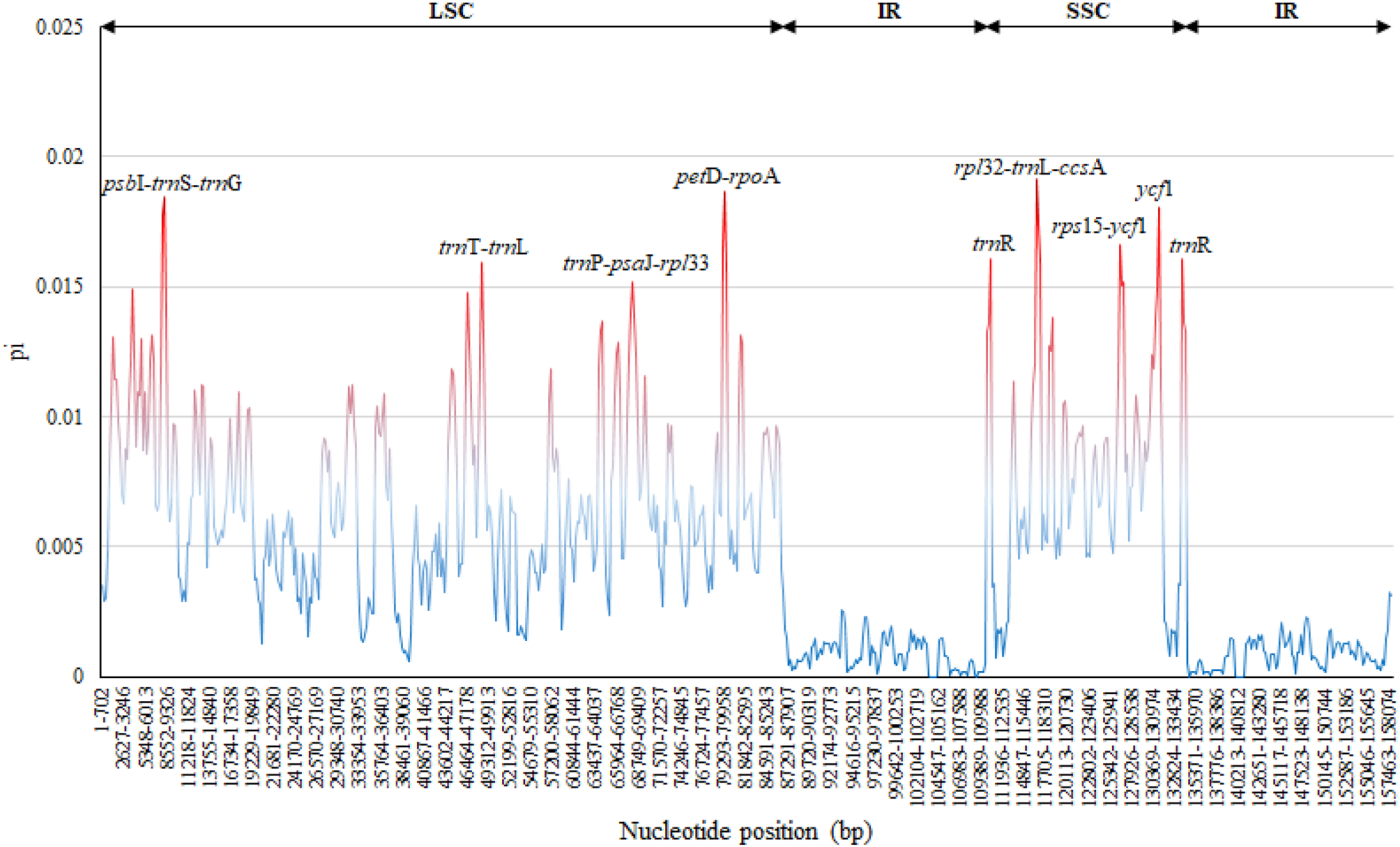
Sliding window analysis of 48 Lilium cp genomes (window length: 600 bp—step size: 200 bp). The X-axis and Y-axis represents the position of a window and nucleotide diversity (Pi) of each window, respectively.

In the *Lilium* species, the IR region was more conserved than LSC and SSC regions. Synteny results revealed that the *Lilium* species have a high degree of sequence identity and collinearity at the cp genome-wide scale, especially in the IR region (Fig. S1). We also conducted genome-wide analysis via sliding window assessment to detect hotspot regions in the *Lilium* cp genomes. Nucleotide diversity (pi) was averaged at 0.00504, ranging from 0 to 0.01913. The variability of *rpl*32-*trn*L-*ccs*A, *pet*D-*rpo*A, *ycf*1, *psb*I-*trn*S-*trn*G, *rps*15-*ycf*1, *trn*R, *trn*T-*trn*L, and *trn*P-*psa*J-*rpl*33 were higher among the 48 *Lilium* cp genomes. The divergence was more prominent in the SC regions than in the IRs regions, which displayed a higher nucleotide variability compared to IR regions (Fig. 3).

### SSRs and complex repeat analysis

**Figure 4:**
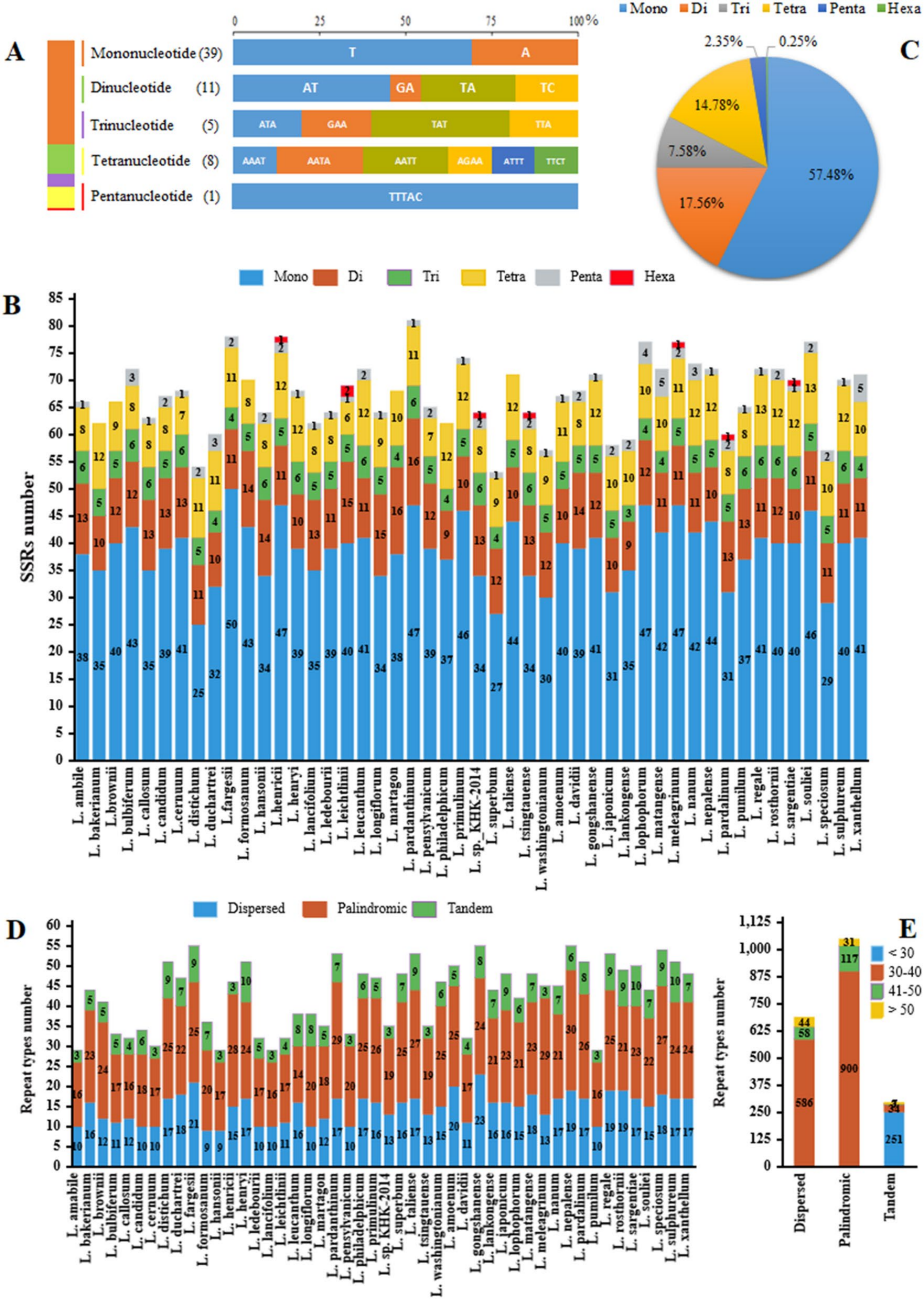
The type and distribution of simple sequence repeats (SSRs) and complex repeat in the 48 Lilium cp genomes. (**A**) Frequency and type of SSRs in the *L. ledebourii* cp genome. (**B**) The number of SSR types discovered in 48 Lilium cp genomes. (**C**) The percentage of SSrs types in 48 Lilium cp genomes. (**D**) The number and of complex repeats types in 48 Lilim cp genomes. (**E**) Frequency of complex repeats by size.

In population genetic studies, the number and position of repeated DNA motifs (with 1–6 nucleotides) have been routinely employed for the detection of polymorphisms in cp genomes^44^. We discovered SSRs in the cp genomes of SCh and 47 closely related species. 64 SSRs were found in the SCh cp genome, mostly (60.94%) made up of mononucleotide repeats. In addition, only one pentanucleotide SSR pattern was observed in SCh (Fig. 4A). As shown in Fig. 4C, among the 48 *Lilium* cp genomes, SSRs ranged from 53 (*L. superbum*) to 81 (*L. pardanthinum*). In total, 3234 microsatellites were detected in 48 cp genomes of *Lilium* (Fig. 4C), with mononucleotide SSRs (57.48%) being the most common, whereas di-, tri-, tetra-, penta-, and hexa nucleotide SSRs accounted for 17.56%, 7.58%, 14.78%, 2.35%, and 0.25% of all SSRs, respectively (Fig. 4B). The number of mono-nucleotide repeats in the 48 *Lilium* cp genomes varied from 25 (*L. distichum*) to 50 (*L. fargesii*). Hexanucleotide repeats were only observed in the cp genome of *L. henricii* (AACTAG/AGTTCT), *L. leichtlinii* (AAATAT/ATATTT and ACTCAT/AGTATG), *L. sp*_KHK-2014 (ACGTAT/ACGTAT), *L. tsingtauense* (ACGTAT/ACGTAT), *L. meleagrinum* (AACTAG/AGTTCT), *L. pardalinum* (AATAGT/ACTATT), and *L. sargentiae* (AAATTC/AATTTG). Overall, varied SSR motifs were found in *Lilium* cp genomes at different frequencies. This research distinguished the presence and SSR types of *Lilium* species, which might be fruitful for molecular marker investigations and population genetics of *Lilium*, especially for *L. ledebourii* (Table S1, Fig. S2).

Complicated repeat sequences play a role in the recombination and variation of chloroplast genomes^45^. The SCh chloroplast genome contains 32 complex repeats, including five tandem, ten dispersed, and 17 palindromic repeats (Table 2). These repeats were at least 24 bp in length, with the longest being 53 bp. Furthermore, it was discovered that the final quantity of complex repeats in the SCh genome was around 25% lower than the average number of repeats in *Lilium* genomes, with a decrease of 18%, 32%, and 22% for tandem, dispersed, and palindromic repeats, respectively (Fig. 4D). In total, 29–55 long repeat sequences were discovered in each *Lilium* cp genome, including 9–23 dispersed repeats, 14–30 palindromic repeats, and 3–10 tandem repeats (Fig. 4D). With the exception of *L. longiflorum*, which contained a repetition of 162 bp, repeat sizes varied from 30 to 87 bp in dispersed, 30 to 61 bp in palindromic, and from 15 to 85 bp in tandem. In other words, out of the 48 species, palindromic repeat was the most common type, and the total number of repeats ranged from 29 to 55, with 60.86% of these repeats being between 30 and 40 in length (Fig. 4E).

**Table 2:** Dispersed and palindromic repeats by positions in the cp genome of *L. ledebourii*.

N	Size (bp)	Start position1	Type	Start position2	E-value
1	53	36,308	D	38,532	5.06E−17
2	43	43,951	P	43,951	1.08E−14
3	38	27,465	P	27,465	8.59E−14
4	34	112,143	P	112,143	2.20E−11
5	40	88,672	D	88,696	3.77E−11
6	40	88,672	P	144,558	3.77E−11
7	40	88,696	P	144,582	3.77E−11
8	40	144,558	D	144,582	3.77E−11
9	39	41,001	D	96,049	1.43E−10
10	39	41,001	P	137,206	1.43E−10
11	41	71,433	P	71,433	3.86E−10
12	35	91,474	P	91,474	5.77E−10
13	35	91,474	D	141,785	5.77E−10
14	35	141,785	P	141,785	5.77E−10
15	34	53,789	P	53,789	1.11E−07
16	35	33,896	P	33,896	9.71E−07
17	34	75,092	D	75,118	3.55E−06
18	31	7496	P	42,412	5.89E−06
19	33	36,334	D	38,558	1.30E−05
20	30	3602	P	68,653	2.20E−05
21	30	5488	P	5488	2.20E−05
22	30	120,861	P	120,896	2.20E−05
23	32	33,306	P	42,412	4.71E−05
24	31	7496	D	33,307	1.71E−04
25	30	9028	D	34,112	6.17E−04
26	30	29,142	D	29,414	6.17E−04
27	30	110,845	P	110,873	6.17E−04

### Codon usage bias analysis

Due to the widespread occurrence of synonymous codon bias in organisms, recognizing codon preference might play a significant role in the evolution by clarifying the selection pressure and improving translation efficiency by utilizing major codons^46,47^. Totally, 21,989 codons were detected in the SCh protein-coding genes. A- and U-ending are seen to be more prevalent than G and C-ending ones. Among SCh amino acids, the highest and lowest frequencies were related to leucine (Leu = 2268) and cysteine (Cys = 255), respectively. In SCh, 30 codons showed more bias (RSCU > 1), and 31 codons displayed bias: RSCU < 1. In addition, there was no bias (RSCU = 1) in the frequency of start codons AUG (methionine), UGG (tryptophan), and AUA (isoleucine) (Table 3).

**Table 3:** The Relative synonymous codon usage (RSCU) of *L. ledebourii* protein-coding genes.

Codon	AA	ObsFreq	RSCU	Codon	AA	ObsFreq	RSCU
UAA	^a^	68	1.468	AUG	M	552	1
UAG	^a^	41	0.885	AAC	N	216	0.422
UGA	^a^	30	0.647	AAU	N	808	1.578
GCA	A	345	1.151	CCA	P	254	1.121
GCC	A	186	0.621	CCC	P	184	0.812
GCG	A	125	0.417	CCG	P	116	0.512
GCU	A	543	1.812	CCU	P	352	1.554
UGC	C	62	0.486	CAA	Q	573	1.504
UGU	C	193	1.514	CAG	Q	189	0.496
GAC	D	175	0.411	AGA	R	401	1.519
GAU	D	676	1.589	AGG	R	127	0.481
GAA	E	859	1.511	CGA	R	298	1.53
GAG	E	278	0.489	CGC	R	81	0.416
UUC	F	435	0.691	CGG	R	109	0.56
UUU	F	824	1.309	CGU	R	291	1.494
GGA	G	588	1.581	AGC	S	86	0.383
GGC	G	171	0.46	AGU	S	363	1.617
GGG	G	253	0.68	UCA	S	352	1.149
GGU	G	476	1.28	UCC	S	259	0.846
CAC	H	108	0.408	UCG	S	144	0.47
CAU	H	421	1.592	UCU	S	470	1.535
AUA	I	647	1	ACA	T	353	1.253
AUC	I	365	0.564	ACC	T	205	0.728
AUU	I	929	1.436	ACG	T	115	0.408
AAA	K	837	1.499	ACU	T	454	1.611
AAG	K	280	0.501	GUA	V	456	1.482
CUA	L	289	1.115	GUC	V	161	0.523
CUC	L	160	0.617	GUG	V	167	0.543
CUG	L	135	0.521	GUU	V	447	1.452
CUU	L	453	1.747	UGG	W	399	1
UUA	L	782	1.271	UAC	Y	162	0.393
UUG	L	449	0.729	UAU	Y	662	1.607

^a^Stop codon.

**Figure 5:**
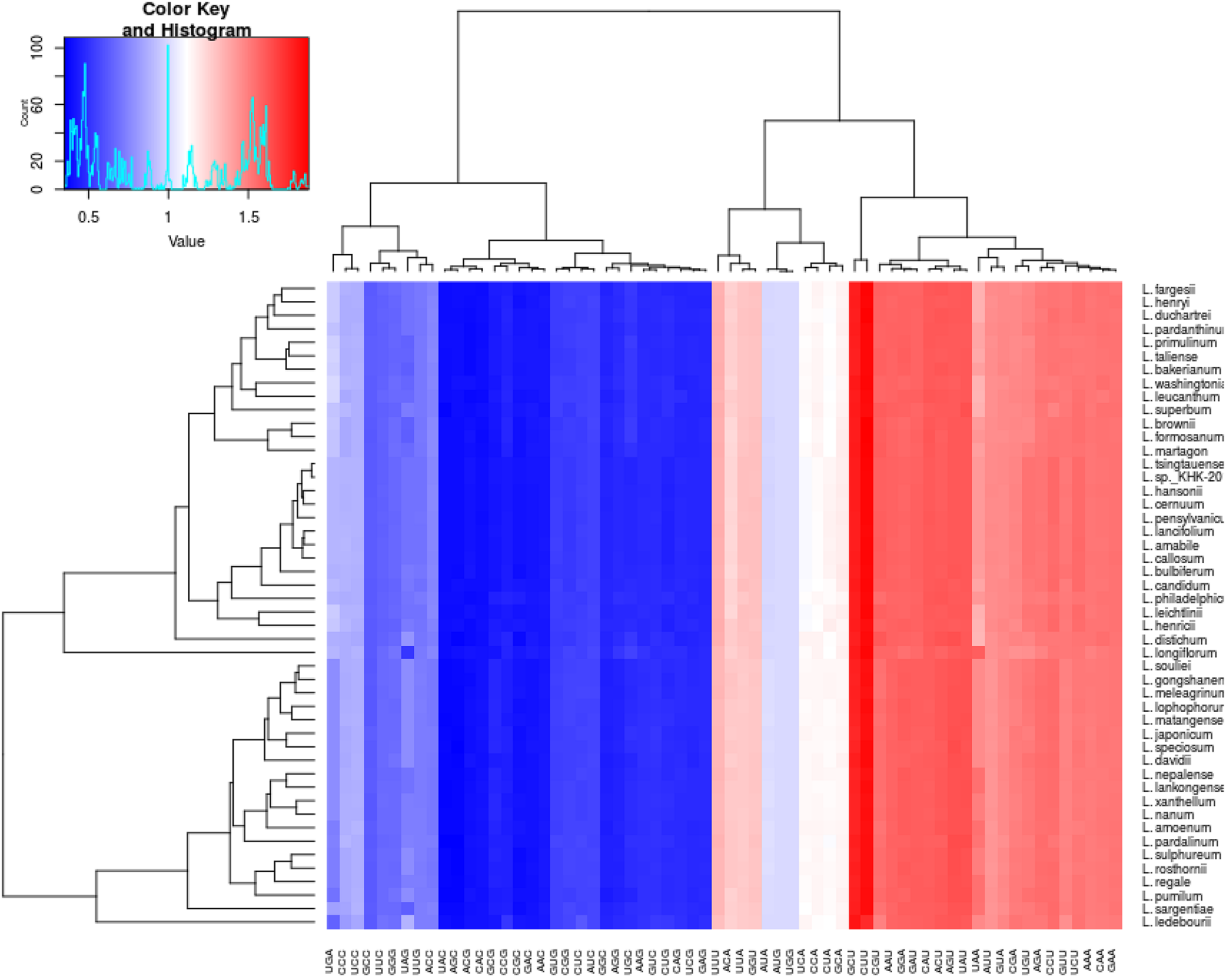
Codon distribution of protein-coding genes among Lilium cp genomes. Color code: Red denotes a higher RSCU and blue denotes a lower RSCU.

Comparing the protein-coding genes in 48 *Lilium* cp genomes, we found that each species was composed of 20,691–22,781 triplet codons in protein-coding genes. Leucine (10.18–10.34%) was the most abundant among encoded amino acids in all of the species studied, whereas cysteine (1.13–1.24%) was the least abundant (Table S2, Fig. 5). Among the 20 amino acids, the lowest and highest RSCU values were recorded for Tyr-UAC, encoding the tyrosine, and Leu-CUU, encoding the leucine, respectively. Codon usage in the *Lilium* cp genomes was biased towards A- and U-ended codons, according to RSCU values (RSCU > 1). In addition, the pattern of codon usage bias in the Lilium species was investigated. Figure S3 shows the values of the Codon adaptation index (CAI), Codon bias index (CBI), Frequency of optimal codons (FOP), Effective number of codons (NC), and GC3s for 48 Lilium chloroplast genomes. We observed that five parameters associated with codon usage bias are very similar across Lilium species (Fig. S3).

### Selection pressure on *Lilium* cp genomes and adaptation evolution

In this study, the Ka/Ks ratio was computed for the 78 protein-coding genes shared by all 48 cp genomes (Table S3). Ks = 0 may lead to swelling of Ka/Ks ratios or misidentification of genes with powerful positive selection because of high, unlimited, or unspecified Ka/Ks ratio. To put down this difficulty, we eliminated the comparisons with Ks = 0 from all analyses. The average Ka/Ks value of the 78 protein-coding genes examined among 48 cp genomes was 0.2472. Among the genes, *ndh*B, *psb*J, *psb*Z, and *ycf*2 exhibit zero synonymous substitution (Ks = 0), revealing divergence of species or genetic restriction. The *rps*7, *atp*H, *pet*N, *psa*I, *psa*J, *psb*F, *psb*L, *psb*T, and *psa*C were the highly conserved genes, with an average Ka/Ks ratio of 0, indicating extremely purifying selection pressure (Table S3). We found that *acc*D, *rpl*16, and *rpl*36 with Ka/Ks average of 1.268, 1.052, and 1.200, respectively, have been subjected to positive selection in the *Lilium* cp genomes. The *mat*K, *pet*B, *pet*D, *rps*4, *rps*12, and *ycf*1 had average Ka/Ks ratios in the 0.5 to 1 range, reflecting calm selection. The Ka/Ks average for the rest genes was recorded less than 0.49, reflecting that about 72% of genes (56/78) in the *Lilium* cp genomes were subject to purifying selection. Although the average Ka/Ks > 1 was recorded only for the *acc*D, *rpl*16, and *rpl*36, for 26 genes, Ka/Ks > 1 was observed in at least one pairwise comparison (from 1128 pairwise comparisons). The gene *ycf*1 possesses 248 positive selective pairwise comparisons, followed by *mat*K (144), *ccs*A (52), *rbc*L (37), *ndh*I (31), *clp*P (29), *atp*F (20), *rpo*C2 (20), *cem*A (16), *ndh*D (14), *ndh*F(10), *pet*B (10), *ndh*A (9), *ndh*G (9), *pet*D (9), *ycf*4 (8), *rpo*A (7), *ndh*J (4), *rpl*33 (3), *rps*14 (3), *ndh*H (2), *pet*G (2), *rps*4 (2), *ndh*C (1), *rpo*B (1), and *rps*2 (1).

**Figure 6:**
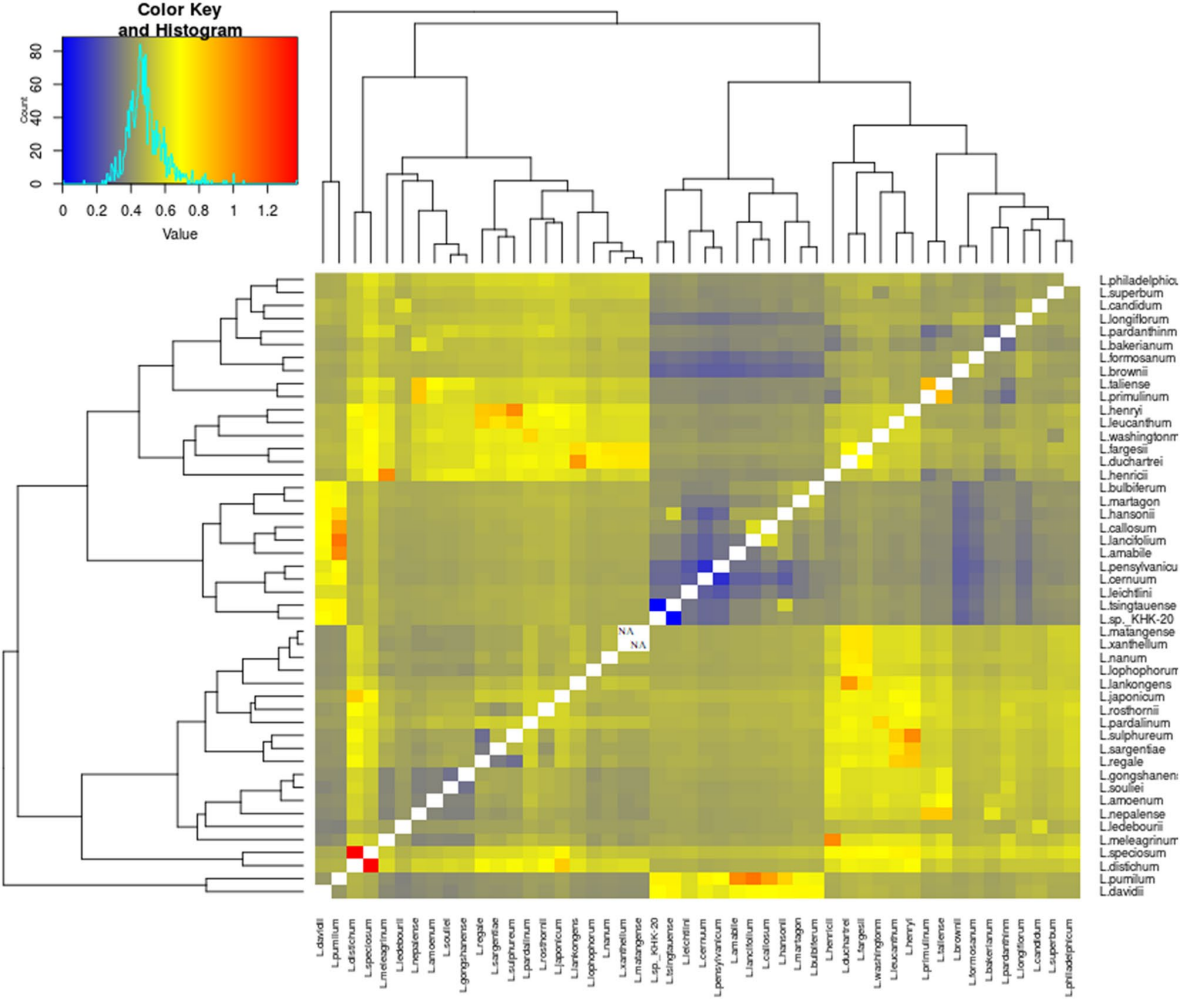
Ka/Ks ratios between Lilium cp genome pairs. In the multigene nucleotide alignment, the heatmap depicts pairwise Ka/Ks ratios between each concatenated single-copy CDs sequence.

Also, another analysis to compare *Lilium* species based on “concatenate all protein-coding genes” was performed and obtained 1128 pairwise comparison outcomes of Ka/Ks values. The pairwise Ks value of zero was seen between *L. matangense* and *L.xanthellum*. Ka/Ks > 1 was only obtained between *L. speciosum* and *L. distichum* with a Ka/Ks ratio of 1.375. The Ka/Ks = 1 was recognized between *L. amabile* and *L. pumilum*, *L. henricii* and *L. meleagrinum*, *L. sulphureum* and *L. henryi*, and *L. lancifolium* and *L. pumilum* (Ka/ks = 1.062). Ka/Ks = 0 among all comparisons, only was recorded between *L. sp.*_KHK-2014 and *L. tsingtauense*. Overall, the average pairwise Ka/Ks value was 0.4839 (Fig. 6), indicating that at the whole cp protein level, *Lilium* species were subjected to a purifying selection.

### Phylogenetic analysis

**Figure 7:**
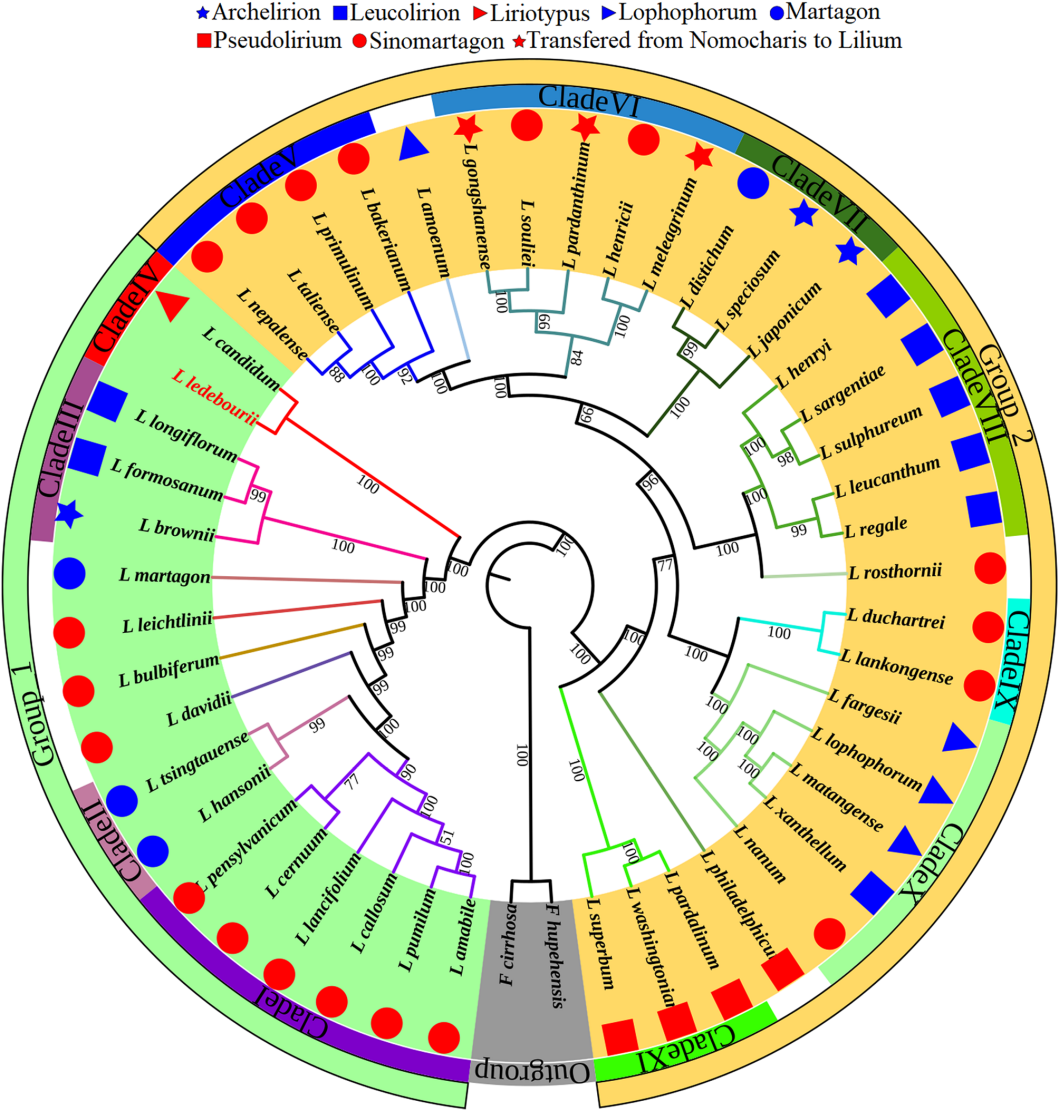
The phylogenetic relationships of Lilium species employing whole cp genome sequences. *Fritillaria hupehensis* and *Fritillaria cirrhosa* were applied as outgroups. Phylogenetic tree were constructed by Maximum likelihood (ML). The ML bootstrap values are represented by the numbers above the branches.

Both analyses, which used the complete chloroplast genome (CCGs) and protein coding genes (CDSs), divided 47 Lilium species into two main groups. Although the members of both main groups were the same in both topologies, there were slight differences between them (Figs. 7, S4). Because of the high synteny among *Lilium* cp genomes, this study concentrated on phylogenetic analysis employing whole cp genome sequences to inspect relationships across the 47 *Lilium* species. Maximum likelihood was employed with two species serving as outgroups. According to the topology, the majority of nodes were highly supported. In all, 34 of the 45 nodes acquired a maximally supported (value ≥ 99%) value bootstrap. According to the CCG topology, the 47 *Lilium* species were divided into two main groups consisting of 11 clades (Fig. 7).

Group 1: included 17 species, which were placed in four clades and four self-sufficient lineages. Clade I consisted of six Sinomartagon species including *L. callosum*, *L. amabile*, *L. cernuum*, *L. lancifolium*, *L. pensylvanicum*, and *L. primulinum*. Clade II is composed of two Martagon species (L*. tsingtauense* and *L. hansonii*). Clade III consisted of two Leucolirion (*L. formosanum* and *L. longiflorum*), and *L. brownie* belong to Archelirion. *L. candidum* of Liriotypus and *L. ledebourii* established a monophyletic clade IV. *L. davidii* was the sister of Claude II. *L. martagon*, *L. leichtlini*, and *L. bulbiferum* had more distant connections with claude I Sinomartagons (Fig. 7).

Group 2: included 30 species, which were placed in seven clades and three self-sufficient lineages. Clade V was composed of four Sinomartagon species including *L. bakerianum*, *L. taliense*, *L. primulinum*, and *L. nepalense*. Clade VI consisted of two Sinomartagon (*L. henricii* and *L. souliei*), and three species (*L. pardanthinum*, *L. gongshanense*, and *L. meleagrinum*), which have recently been transferred from the genus Nemacaris to the genus *Lilium*. *L. speciosum*, *L. japonicum*, and *L. distichum* formed calde VII. Five species of Leucolirion including *L. regale*, *L. leucanthum*, *L. sulphureum*, *L. sargentiae*, and *L. henryi*, created clade VIII. Clade IX accommodated *L. duchartrei* and *L. lankongense*. Three species of Lofoforum (*L. fargesii*, *L. lophophorum*, and *L. matangense*), along with *L. nanum* and *L. xanthellum*, were included in clade X. Three species (*L. superbum* and *L. washingtonianum*, and *L. pardalinum*) belonging to Pseudolirium formed clade XI. *L. amoenum*, *L. rosthornii*, and *L. philadelphicum* were sisters to the clade V, clade VIII, and clade X, respectively (Fig. 7).

## Discussion

The conserved characteristics of gene content and organization, as well as the GC content of the SCh cp genome, were found to be similar to the variability in other species^17,18^. Furthermore, trans-splicing was observed in the *rps*12 gene, which is also seen in other species^48^. The *Lilium* cp genomes length differed between 151,655 bp in *L. bakerianum* and 153,235 bp in *L. fargesii*. It was suggested that one of the primary causes for the change in the cp genomes size is that the IR region shrinks, expands, or losing^49^.

We surveyed the expansion and contraction variability in IR/SC junction regions. The boundary of LSC/IRb is stable, while slightly visible junction variation can be seen in the IRa/LSC and IRb/SSC boundaries. The occurrence of contraction and expansion of IR regions during evolution is a relatively common happening, which has been employed as evolutionary loci for phylogenetic studies^50,51^. Expansion of IR/LSC junctions to *rps*19 has been observed in other Liliaceae species such as Amana, and this event appears to be a Liliaceae ancestral symplesiomorphy^52^. The contraction/expansion of IR regions in Liliaceae has resulted in the formation of *ycf*1 and *rps*19 at the boundaries across SC and IR regions, with varying lengths, as demonstrated in *Fritillaria*^53^ and *Cardiocrinum*^54^. Given the unison of our findings with those about other plants^52–54^, expansion and contraction of IR regions may be a significant mechanism for different lengths of 48 *Lilium* cp genomes.

Analysis of nucleotide diversity and cp repeats can be used to recognize molecular markers, rebuild evolutionary connections, and delve into population genetics^55,56^. In this study, a total of 3234 microsatellites were discovered in 48 cp genomes of *Lilium*. A/T repeats were the most common type of SSR found. The abundance of this SSR type is consistent with the majority of other cp genomes explored thus far^57^. Additionally, complex repeats in the 48 *Lilium* were found, which could be substantial genomic reconfiguration hotspots^58,59^. The number and size of tandem, dispersed, and palindromic repeats were nearly identical in the cp genomes of relevant species such as *Fritillaria*^60^. Especially, the incidence of large repeats in the chloroplast, such as the 162 bp tandem repeat of *L. longiflorum* from our results, has probably been linked to an unstable genomic structure because of improper rearrangements^61^.

Here, a higher divergence of the SC regions and a lower divergence of the IR were discovered, suggesting that the IR is more conservative than in other regions, with the same characteristics of most angiosperms^62^. This occurrence is caused by copy correction of IRs and the removal of harmful mutations via gene conversion^63^. *rpl*32-*trn*L-*ccs*A^64^, *trn*P-*psa*J-*rpl*33, *pet*D-*rpo*A, *ycf*1^18^, *psb*I-*trn*S-*trn*G^65^, and *rps*15-*ycf*1, *trn*T-*trn*L^66^ have previously been identified as high variability regions in various species. The phylogeny of genus *Lilium* will be clarified with the help of these regions, which are expected to be very helpful in the future.

The rate of Ka (non-synonymous) to Ks (synonymous) nucleotide substitution is commonly employed as a powerful tool for the clarification of the evolution of protein coding genes and also species adaptive developments^67,68^. The Ka/Ks ratio determines the gene divergence grade and whether selection pressure is positive (Ka/Ks > 1), purifying (Ka/Ks < 1, particularly if it is less than 0.5), or neutral (Ka/Ks = 1)^67^.

In the present study, the Ka/Ks > 1 was recorded for *acc*D, *rpl*16, and *rpl*36, implying that these genes could be important in the adaptive evolution^51^. Positive selection of *acc*D was signed by the important role of the gene in stress tolerance and resistance, insect predation, and pathogens^69^. Positive selection in *acc*D has been observed in Ipomoea^41^ and Stauntonia^43^. Other positively selected genes were *rpl*16 and *rpl*36, which are responsible for encoding the ribosomal protein, which has been evidenced to be necessary for the development of chloroplast ribosomes in plants^70^. Previous studies have reported positive selection for *rpl*16 in Lonicera^71^ and *rpl*36 in Aquilegia^42^.

However, we discovered that some genes were positively selected in at least one pairwise comparison, suggesting these genes were potentially subject to positive pressure for selection among *Lilium* species. *ycf*1 possesses 248 positive selective pairwise comparisons, followed by *mat*K (144), *ccs*A (52), *rbc*L (37), *ndh*I (31), *clp*P (29), *atp*F (20), *rpo*C2 (20), *cem*A (16), *ndh*D (14), *ndh*F(10), *pet*B (10), *ndh*A (9), *ndh*G (9), *pet*D (9), *ycf*4 (8), *rpo*A (7), *ndh*J (4), *rpl*33 (3), *rps*14 (3), *ndh*H (2), *pet*G (2), *rps*4 (2), *ndh*C (1), *rpo*B (1), and *rps*2 (1). Of these, *Mat*K has previously been found under positive selection in over 30 different taxonomic groups^72^. NADH-dehydrogenase gens group (*ndh*) were fundamental in the use of light energy and the electron transfer chain to produce ATP, significant components for photosynthesis^73,74^. As a consequence, these genes, as important components involved in plant growth, may have evolved as a result of more common substitutions in order to adapt to various environmental conditions^43^. We discovered positive selection on the *ycf*1 gene in 248 pairwise comparisons. The *ycf*1 is huge open reading frame, which encodes protein products for many amino acids in higher plant. Moreover, the necessity of the *ycf*1 gene for cell survival has been proven by knockout studies^75^. *clp*P, which encodes the (ATP-dependent) clp protease, is thought to play a role in chloroplast protein transformation and may be necessary for shoot development in the presence of the degradation of clpP-mediated protein^76,77^. Another positively selected gene in our study is the rubisco large-chain gene (*rbc*L). In many higher plants, *rbc*L positive selection has been made^78^. *atp*F is involved in the encoding of the H^+^-ATP subunits, which is necessary for some photosynthetic processes^79^. Positive selection has been noticed to evolve *rpo* genes, which encode proteins role-playing in the modification of transcription and post-transcriptional modification^80^. Due to the cooperation of *cem*A with nuclear genes^81^, cemA might evolve relatively quickly in species^82^. Substitution of amino-acid, indel presence and prematurity in stop codon could lead to a positive selection of *ccs*A^83^.

Selected positive genes may have had crucial roles in the adaptation of *Lilium* to different environments. Gao et al.^84^ documented the adaptation of chloroplast genes to various ecological environments of solar preferences. Moreover, more undiscovered selective compulsions may be involved in the increasing of the Ka/Ks ratio, leading to species divergence^85^. The Ka/Ks ratios in the majority of genes shared by *Lilium* chloroplast genomes and among pairwise comparisons of species employing all protein-coding genes were less than 1, proposing purifying selection. Similar findings were detailed for *Gentiana* species^86^. This lower rate of Ka/Ks can be the result of the fact that most of the species are probably to undergo disadvantageous nonsynonymous substitutions and purification selections, and the selective restriction on nonsynonymous substitutions is stronger than synonymous substitutions^87,88^. In short, positive selection in some genes likely enriches the *Lilium* variety and adaptability.

At the whole cp protein scale, *Lilium* species were subjected to a purifying selection. Sunlight UV radiation damages and rearranges DNA^89,90^, and higher temperatures speed up metabolism^91^, all contributing to an increase the mutation rates. Consequently, purifying selection, as one of the most common types of natural selection, constantly helps in the elimination of disadvantageous mutations in populations. Purifying selection would thus be an evolutionary outcome of the preservation of *Lilium* species adaptive habits.

The richer the taxon sampling, the more accurate it is to comment on the Comber’s classification. The present study clustered 47 species of *Lilium*. To date, some of them have not been evaluated at the whole cp genome level. *Lilium* species were distributed into 11 clades divided into two main groups. The position of the species in the present topology is consistent with the classification by Kim et al.^12^.

Our samples did not support the monophyly of Comber’s sections^11^. According to our results, *L. martagon* was placed farther away from the Martagon species and was closer to *L. leichtlinii*, *L. bulbiferum*, and *L. davidii,* which agrees with Gong et al.^16^ based on nrITS. We observed *L. brownii* of Comber’s Archelirion far from the other two species of Archelirion*) L. japonicum* and *L. speciosum*( and close to the two species of Leucolirion (*L. formosanum* and *L. longiflorum*). Similarly, Li et al.^9^ classified *L. brownii* alongside *L. formosanum* and *L. longiflorum* in a genus-level study of the Liliaceae family's evolution. As our results warn, and with the help of ITS-dataset classification^92,93^, now with the approval of cp genome-based classification, *L. brownii* can be moved from the Archelirion to the Leucolirion.

Based on the morphology, *L. duchartrei*, *L. lankongense*, *L. nanum*, and *L. rosthornii* belong to the Sinomartagon section^11^. However, based on our cp genome-scale topology, these species are further away from the Sinomartagons and are closer to the Lophophorum species. Studies have shown that *L. nanum* and Lophophorum have similar karyotypes^94^. Additionally, according to the ITS regions, Du et al.^92^ reported that *L. duchartrei* and *L. lankongense* are in the same clade and are closer to the Lophophorum. We accommodated *L. xanthellum* on Clade X, away from Leucolirion. According to ultrametric chronograms, *L. xanthellum* is closer to *L. lophophorum* and *L. matangense*^95^. Totally, the composition of clade X in our phylogeny (three species of Lophophorum including *L. fargesii*, *L. lophophorum*, and *L. matangense*, along with *L. nanum* of Sinomartagon and *L. xanthellum* of Leucolirion) is in agreement with Du et al.^92^ based on ITS regions.

Although, the monophyletic of the three Martagon species (*L. hansonii*, *L. tsingtauense*, and *L. distichum*) were rejected^12,92^ prior to our study, so far, the position of *L. distichum* has not been clear enough due to restriction of the sampling of Lophophorum and Nomocharis. Based on our topology tree and with the help of a richer sampling, *L. distichum* is further away from its companions in previous studies. Our phylogenetic tree shows *L. distichum* close to two species of Archelirion (*L. japonicum* and *L. speciosum*). Moreover, in terms of flower morphology, Comber’s Martagon members have differences from each other. The flowers of *L. distichum* are outfacing, whereas those of *L. tsingtauense* and *L. martagon* are upright and nodding, respectively^19^.

As shown in the results (Fig. 7), *L. henricii* and *L. souliei*, two species of Comber’s Sinomartagon, are placed next to Nomocharis-Lilium (*L. gongshanense*, *L. meleagrinum*, and *L. pardanthinum*). Gao et al.^96^ based on biogeographic results, showed that *L. henricii* is associated with Nomocharis species. What is more, Gao et al.^97^ by examining 38 *Lilium* species and 7 Nomocharis species using the ITS dataset, showed *L. souliei* inside the Nomocharis clade.

Based on our CCG topology, *L. amoenum* of Lophophorum was sister to *L. bakerianum*, a member of clade V. Zhou et al.^98^, based on fluorescence in situ hybridization, showed the signal pattern of 35S rDNA in *L. amoenum* was the same as in *L. bakerianum*. Moreover, these researchers, by mapping the chromosome pattern for 35S rDNA based on ITS data, showed that these two species are monophyletic.

The phylogenetic position of *L. ledebourii* was very ambiguous due to the scarcity of molecular information. To date, two studies have attempted this. Kim and Kim^8^ involved *L. ledebourii* (with 15 other species) in building the phylogenetic tree. However, this research does not provide a clear picture of the position of this species, due to sampling restriction and the use of only four chloroplast genes (*rbc*L, *mat*K, *ndh*F, and *atp*B), which according to the genome-scale results, are not among the most divergent hotspots. Ghanbari et al.^99^, based on the ITS marker, have examined the position of this species and shown that *L. ledebourii* (Damash sample) is far from the *L. candidum*.

Following the resolution of *L. ledebourii*, as one of the study's objectives, and according to Kim et al.^12^, who reported that *L. candidum* position remained uncertain, interestingly here, according to cp genome-scale comparisons, *L. ledebourii* and *L. candidum* were monophyletic. *L. ledebourii* is a rare species that has only been seen in Iran and Azerbaijan^20^. Furthermore, *L. candidum* is thought to have originated in Persia and Syria^100^. In total, our findings indicate that a whole cp genome phylogenomic comparison would resolve much controversy and pave the way for *Lilium* phylogeny, especially for *L. ledebourii*.

## Conclusions

The whole chloroplast genome of *L. ledebourii* is reported for the first time. The current study using whole chloroplast genomes of *Lilium* revealed structural characteristics, sequence diversity, and enhanced links between species. Meanwhile, certain variation hotspots identified as high variability regions could function as particular DNA barcodes. We provide a comprehensive analysis of selection pressure, and in the whole cp protein scale, *Lilium* species were subjected to a purifying selection. This study covered the restriction of sampling of Lophophorum and Nomocharis as much as possible. For the first time, *L. ledebourii* participated in the classification of genus *Lilium*, and its position was determined. The position of some species, e.g., *L. distichum*, became clearer than before. It is suggested that *L. brownii* can migrate from the Archelirion to the Leucolirion. We believe that the *Lilium* species have been classified with more excellent resolution than in earlier studies, which will be helpful in the understanding of the evolution of *Lilium* species. The genetic resources provided here will aid future studies in species identification, population genetics, and *Lilium* conservation.

## Materials and methods

### Sample collection and DNA extraction

Fresh leaves of *L. ledebourii* were sampled from Damash village and frozen in liquid nitrogen. The leaf samples were gathered in compliance with national and international legislation and guidelines. It was certified by the herbarium of the Faculty of Agricultural Science and Engineering, University of Tehran, and a validated voucher specimen was deposited at the Department of Horticultural Science, with voucher specimen number 6594. The total genomic DNA was isolated from leaves utilizing a DNeasy plant DNA extraction kit (Qiagen, USA) and the manufacturer's guidelines. DNA integrity was evaluated applying 1% agarose gel, and DNA quantification was assessed employing a NanoDrop spectrophotometer. Extracted DNA was stored at − 80 °C.

### Chloroplast DNA sequencing and genome assembly

SMRT library with a 15–20 Kb insert size was sequenced applying the PacBio RS II platform in Duke Center for Genomics and Computational Biology, USA. To extract potential chloroplast sequences, the PacBio data were mapped to the reference *L. hansonii* cp genome (KM103364) data using BLASR^101^. Error correction was performed on SMRT reads by sprai pipeline^102^. The corrected reads were assembled employing Perl-based pipeline^103^. Furthermore, overlapping ends were checked by “check_circularity.pl” script.

### Chloroplast genome annotation

GeSeq^104^, with NCBI RefSeq for *Lilium* as the reference dataset, was utilized for the annotation of protein-coding, ribosome RNA (rRNA), and transfer RNA (tRNA) genes. Using BLAST against cp genes, sequence coordinates of all annotated genes were checked and manually edited. The tRNAscan-SE version 2.0 was used to double-check the tRNA genes^105^. A circular physical map of the chloroplast genome was illustrated using OrganellarGenomeDRAW (OGDRAW) toolkit^106^ and Chloe (https://github.com/ian-small/chloe).

### Repeat sequence analysis

The MicroSAtellite identification tool (MISA) was applied to screen simple sequence repeats (SSRs) in 48 cp genomes with a threshold of 10 for mononucleotide simple sequence repeats (SSRs), 5 for di-, 4 for tri-, and 3 for tetra-, penta-, and hexa nucleotide^107^. Tandem repeats were also discovered using default parameters by Tandem Repeats Finder v4.09^108^ using default parameters. Moreover, Vmatch V2.3.1^109^ was used to identify palindromic repeats (≥ 20 bp) and dispersed (≥ 30 bp).

### Chloroplast genome comparison

In order to discover the *Lilium* divergence regions, the distance among adjoining genes and junctions of *L. ledebourii* SSC, LSC, and IRs regions, were compared to the other Lily cp genomes species. For each particularized codon, the ratio of usage frequency was obtained as the Relative Synonymous Codon Usage (RSCU) value using DAMBE V6 for 48 *Lilium* species^110^. We mapped the results of codon preference via the R program. To evaluate the degree of codons bias, the CodonW V1.4.4 (http://codonw.sourceforge.net) calculated the values of the codon bias index (CBI), the codon adaptation index (CAI), the frequency of optimal codons (Fop), the effective number of codons (NC), and the GC content of synonymous third codon positions (GC3s)^111^. To discover mutation hotspot sites, the nucleotide diversity in the *Lilium* chloroplast genomes was quantified employing sliding window analysis via DnaSP 6 software^112^. The window length and step size were fixed at 600 bp and 200 bp, respectively.

### Selection pressure on *Lilium* cp genomes

We extracted the CDS sequences of the protein-coding genes from all 48 species, and flushed out those with lacking data in at least one species, which resulted in 78 CDS matrices. MAFFT v7^113^ was used to generate CDS alignments. DnaSP 6^112^ was used to compute the rates of nonsynonymous (Ka) and synonymous (Ks) nucleotide substitution. The selective pressure was measured using the Ka/Ks ratio, with Ka/Ks < 1, Ka/Ks = 1, and Ka/Ks > 1, indicating purifying, neutral, and positive selection, respectively^67^.

### Phylogenetic analysis

The MAFFT v7 program was employed to align the complete cp genome sequence of 47 *Lilium* species^113^. Two complete sequences of *Fritillaria hupehensis‌* and *Fritillaria cirrhosa*‌‌ were applied as outgroups. Utilizing the effective nucleotide substitution model (GTR + G), the maximum likelihood was carried out with RAxML v8.2.11^114^ with 1000 bootstrap repetitions to construct the phylogenetic tree. Finally, the iTOL tool was employed for visualizing the coming about phylogenetic tree^115^. Furthermore, phylogenetic analyses were also carried out for the single-copy protein coding genes (CDSs) shared by all 47 species.

## Supplementary Information

Supplementary Legends.

Supplementary Figure S1.

Supplementary Figure S2.

Supplementary Figure S3.

Supplementary Figure S4.

Supplementary Table S1.

Supplementary Table S2.

Supplementary Table S3.
