# Supplementary material for: Complete chloroplast genome of Lilium ledebourii (Baker) Boiss and its comparative analysis: lights into selective pressure and adaptive evolution
Source: Sci Rep. 2022 Jun 7;12:9375. doi: 10.1038/s41598-022-13449-x (PMC9174193; doi:10.1038/s41598-022-13449-x)
Supplement: Supplementary file 1 — Supplementary Legends. [file 41598_2022_13449_MOESM1_ESM.docx]

Supplementary Information

Complete chloroplast genome of *Lilium ledebourii* (Baker) Boiss and its comparative analysis: Lights into selective pressure and adaptive evolution

Morteza Sheikh-Assadi^1*^, Roohangiz Naderi^1*^, Mohsen Kafi^1^, Reza Fatahi^1^, Seyed Alireza Salami^1^ and Vahid Shariati^2^

^1^Department of Horticultural Science, Faculty of Agricultural Science and Engineering, University of Tehran, Karaj, Iran. ^2^NIGEB Genome Center, National Institute of Genetic Engineering and Biotechnology, Tehran, Iran.

Supplementary Figure 1.

Synteny and rearrangements among in Lilium cp genomes. Color-coded syntenic homologies were connected by lines. The degree of sequence similarity is represented by histograms within each block.

Supplementary Figure 2.

Presence and SSR motifs were distinguished among Lilium cp genomes. Color code: Red denotes a higher frequency and blue denotes a lower frequency.

Supplementary Figure 3.

The comparison of codon usage bias among Lilium species. (A): CAI (Codon adaptation index), (B): CBI (Codon bias index), (C): FOP (Frequency of optimal codons index), (D): ENC (Effective number of codons), (E): GC3s (GC of synonymous codons in 3rd position).

Supplementary Figure 4.

The phylogenetic relationships of Lilium species employing protein-coding genes CDs sequences. *Fritillaria hupehensis‌* and *Fritillaria cirrhosa* were applied as outgroups. Phylogenetic tree were constructed by Maximum likelihood (ML). The ML bootstrap values are represented by the numbers above the branches.

Supplementary Table 1.

Frequency of classified repeat types among the 48 Lilium cp genomes

Supplementary Table 2.

The Relative synonymous codon usage (RSCU) in 48 Lilium cp genomes. *Stop codon.

Supplementary Table 3.

The average of Ka/Ks ratios of 78 protein-coding regions (CDS) within 48 Lilium cp genome.
