# Supplementary figures and images for: Complete chloroplast genome of Lilium ledebourii (Baker) Boiss and its comparative analysis: lights into selective pressure and adaptive evolution
Source: Sci Rep. 2022 Jun 7;12:9375. doi: 10.1038/s41598-022-13449-x (PMC9174193; doi:10.1038/s41598-022-13449-x)

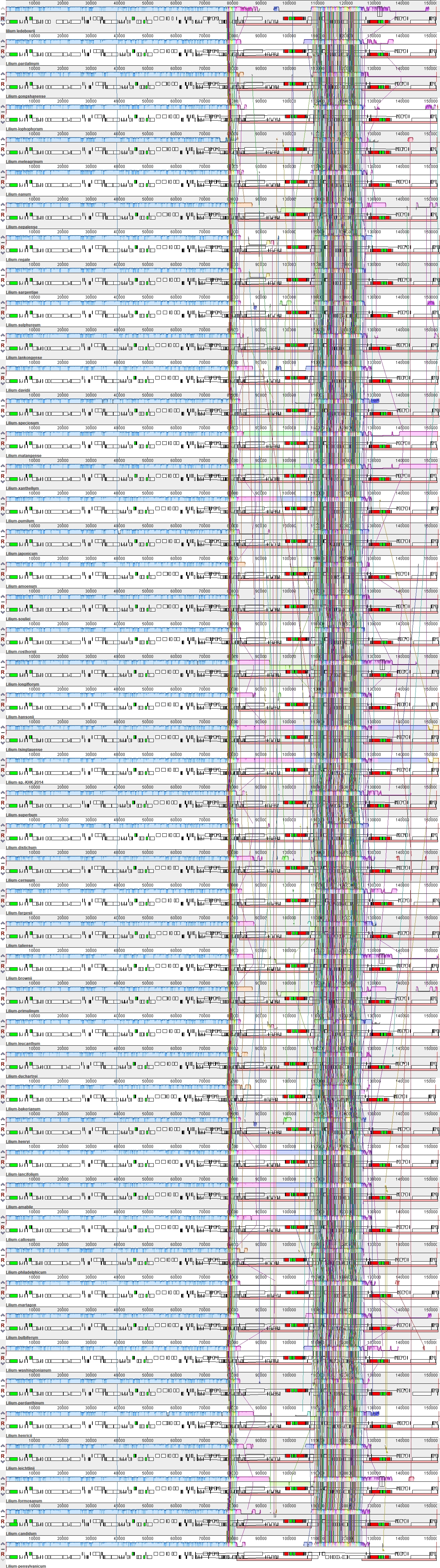

Supplement: Supplementary file 2 — Supplementary Figure S1. [file 41598_2022_13449_MOESM2_ESM.jpg]

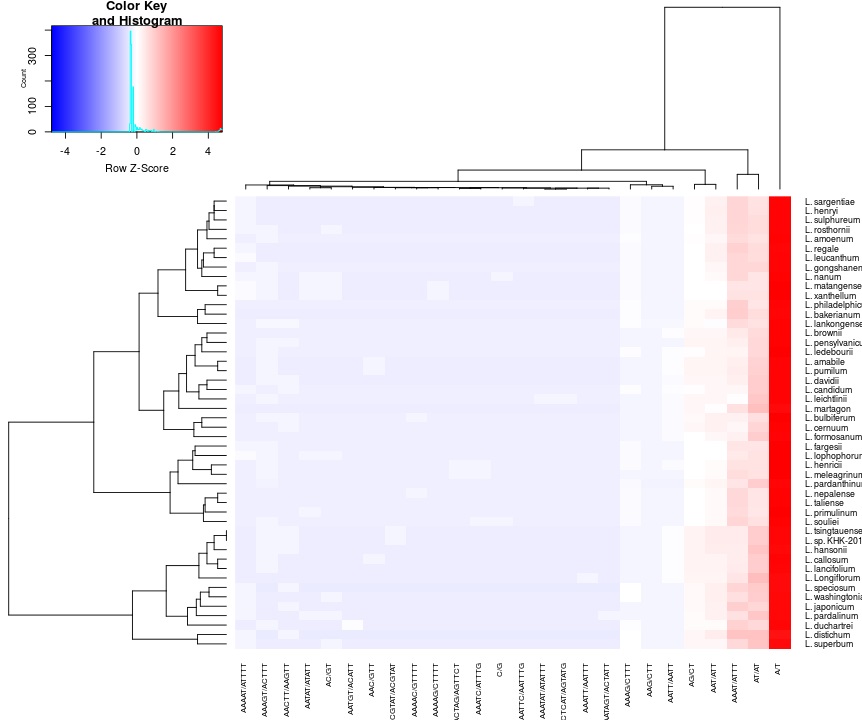

Supplement: Supplementary file 3 — Supplementary Figure S2. [file 41598_2022_13449_MOESM3_ESM.jpg]

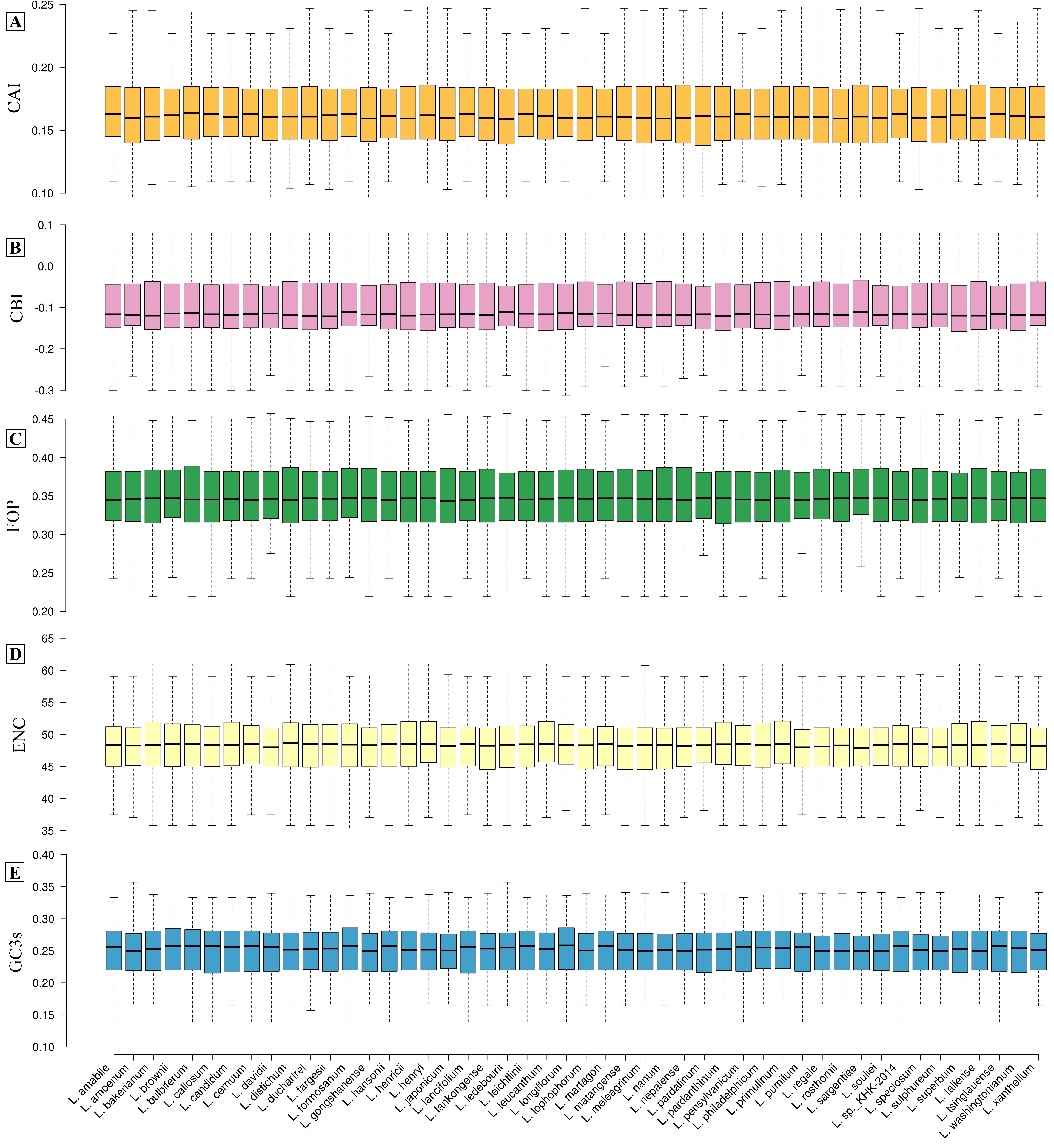

Supplement: Supplementary file 4 — Supplementary Figure S3. [file 41598_2022_13449_MOESM4_ESM.jpg]

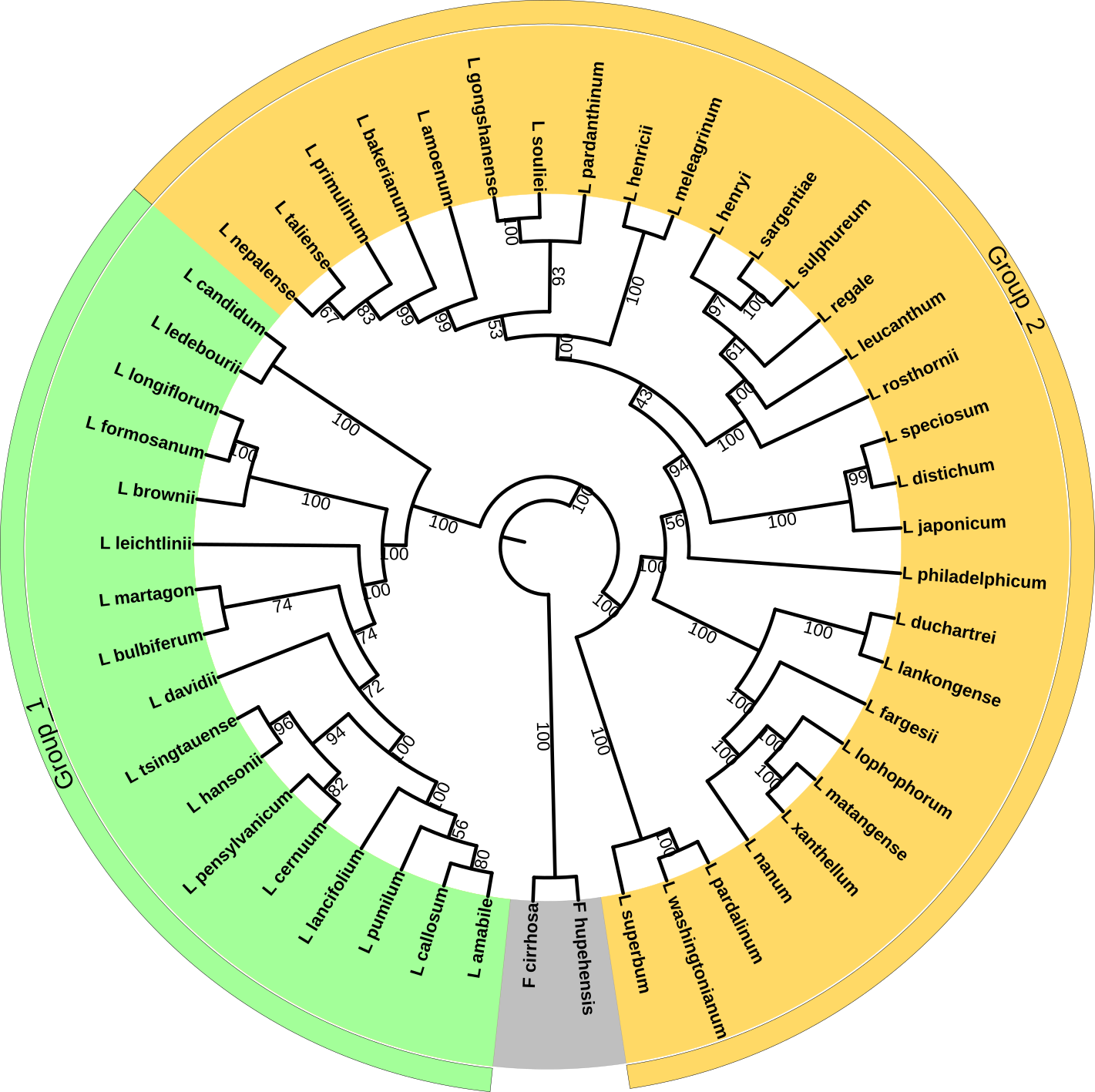

Supplement: Supplementary file 5 — Supplementary Figure S4. [file 41598_2022_13449_MOESM5_ESM.png]
